# Extracellular vesicles derived from renal cancer stem cells induce a pro-tumorigenic phenotype in mesenchymal stromal cells

**DOI:** 10.18632/oncotarget.3503

**Published:** 2015-03-10

**Authors:** Rafael Soares Lindoso, Federica Collino, Giovanni Camussi

**Affiliations:** ^1^ Department of Medical Sciences and Molecular Biotechnology Center University of Torino, Torino, Italy; ^2^ Institute of Biophysics Carlos Chagas Filho, Federal University of Rio de Janeiro, Rio de Janeiro, Brazil; ^3^ Translational Center of Regenerative Medicine Unito/Fresenius Medical Care, Torino, Italy

**Keywords:** extracellular vesicles, cancer stem cells, mesenchymal stromal cells, tumor growth, renal carcinoma

## Abstract

Renal carcinomas have been shown to contain a population of cancer stem cells (CSCs) that present self-renewing capacity and support tumor growth and metastasis. CSCs were shown to secrete large amount of extracellular vesicles (EVs) that can transfer several molecules (proteins, lipids and nucleic acids) and induce epigenetic changes in target cells. Mesenchymal Stromal Cells (MSCs) are susceptible to tumor signalling and can be recruited to tumor regions. The precise role of MSCs in tumor development is still under debate since both pro- and anti-tumorigenic effects have been reported. In this study we analysed the participation of renal CSC-derived EVs in the interaction between tumor and MSCs. We found that CSC-derived EVs promoted persistent phenotypical changes in MSCs characterized by an increased expression of genes associated with cell migration (*CXCR4*, *CXCR7*), matrix remodeling (*COL4A3*), angiogenesis and tumor growth (IL-8, Osteopontin and Myeloperoxidase). EV-stimulated MSCs exhibited *in vitro* an enhancement of migration toward the tumor conditioned medium. Moreover, EV-stimulated MSCs enhanced migration of renal tumor cells and induced vessel-like formation. *In vivo,* EV-stimulated MSCs supported tumor development and vascularization, when co-injected with renal tumor cells. In conclusion, CSC-derived EVs induced phenotypical changes in MSCs that are associated with tumor growth.

## INTRODUCTION

The tumor is composed by different cell populations. A subset of tumor cells, defined as cancer stem cells (CSCs), are characterized by self-renewal and continuous proliferation capacity, providing the ability to initiate tumor and to generate other heterogeneous cell populations [1]. Tumor survival and growth are associated with the capacity of cancer stem cells and of their progenies to interact with the surrounding stromal cells [2]. The mechanisms involved in this crosstalk are based on cell-to-cell contact and on paracrine secretion of several molecules including growth factors, cytokines and inflammatory mediators [3]. In addition, tumor cells interaction were also shown to be mediated by secretion of extracellular vesicles (EVs) that can transfer proteins, lipids and nucleic acids and induce epigenetic changes in target cells [4,5]. This EV communication is associated with cancer development, chemo-resistance and capacity of escaping from immune surveillance [6-9]. Numerous reports showed that tumor EVs are not restricted only to tumor microenvironment but are also present in the blood circulation and other body fluids, supporting the idea that EVs can also stimulate cells at distant sites in the organism [10-13].

Bone marrow-derived mesenchymal stromal cells (MSCs) are known by their migratory capacity to injury and tumor sites. These cells are susceptible to signaling molecules secreted by the tumor and tumor-related stromal cells, promoting their recruitment from bone marrow into the circulation and subsequent engraftment within tumor microenvironment [14]. The role played by MSCs after incorporation in the tumor is still under debate. Anti-tumoral effects have been reported in different types of tumors, showing a reduction in tumor growth and metastasis with the presence of MSCs or EVs derived from these cells [15-18]. However, other studies indicated a pro-tumorigenic role of MSCs, supporting angiogenesis and tumor aggressiveness [19-22]. Such dual role possibly depends on a complex mechanism of interaction between tumor cells and MSCs that can be initially triggered by tumor EVs. Peinado *et al.* showed that bone marrow progenitor cells can be “educated” by exosomes secreted by melanoma to increase tumor metastasis [23].

Our group previously demonstrated the presence of a CSC population in renal carcinoma that releases EVs that favor tumor growth by increasing vascularization and development of a pre-metastatic niche [6, 24]. In the present study, we aimed to analyze whether EVs derived from CSCs (CSC-EVs) induce phenotypical changes in MSCs that favor tumor progression.

## RESULTS

### Incorporation of CSC-EVs in MSCs

**Figure 1:**
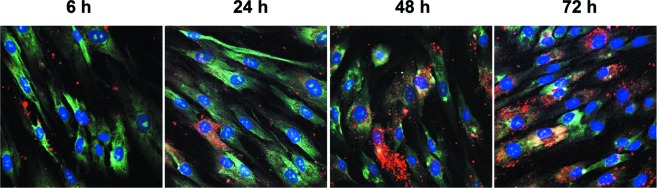
Uptake of CSC-EVs by MSCs CSC-EVs stained with Vybrant Dil (in red) were incubated for 6, 24, 48 and 72 hours with MSCs. Cytoplasm staining of MSCs was obtained by using Syto-RNA dye (in green). The nuclei of MSCs were stained with DAPI (in blue). The different times of incubation are identified. Images were obtained with original magnification × 60. Images are representative of three different experiments.

CSC-EV uptake by MSCs was assessed by maintaining cells incubated with stained CSC-EVs for different periods of time. As shown in Fig. 1, CSC-EVs (in red) were detectable within MSCs already at 6 hours to increase thereafter up to 72 h. Experiments were performed in order to asses population doubling time on MSCs stimulated with CSC-EVs and unstimulated MSCs. No change in population doubling time was observed between the two groups (data not shown).

### CSC-EVs increased MSC migration

**Figure 2:**
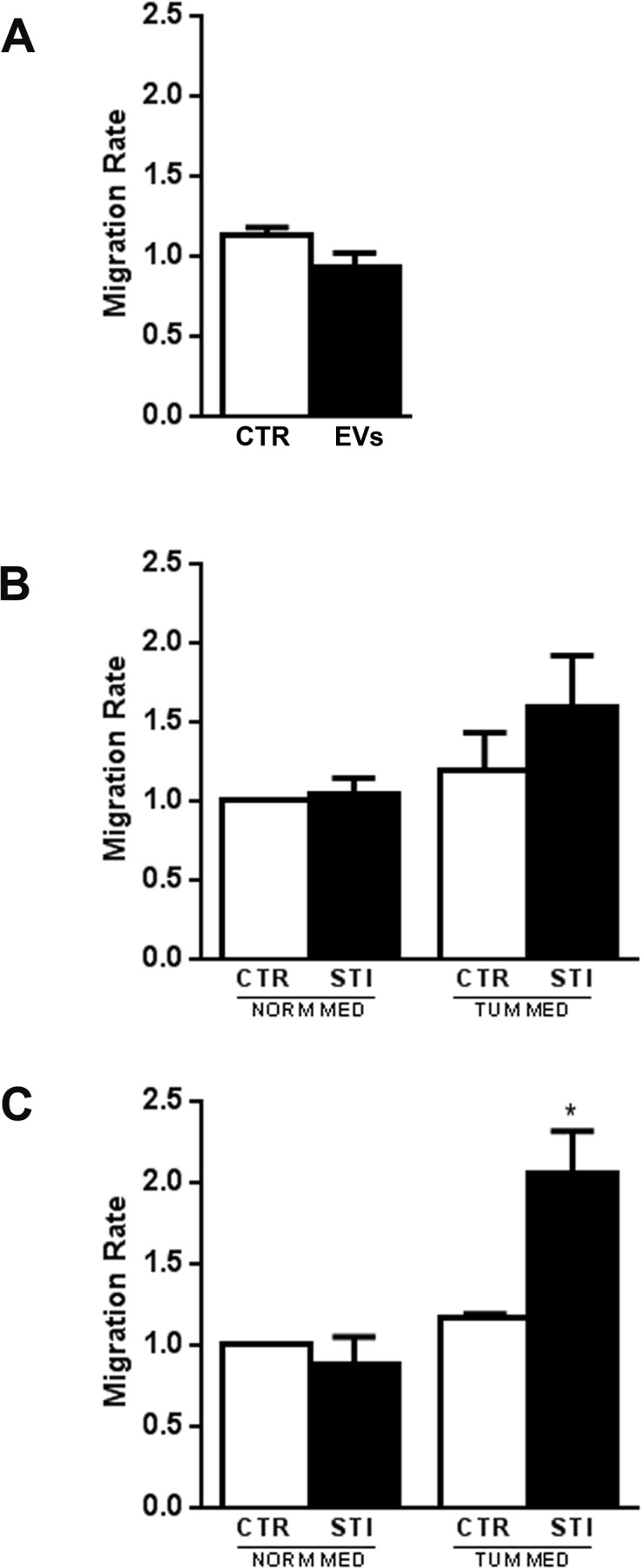
MSC migration after CSC-EV stimulation (A) chemoattraction potential of CSC-EVs was assessed by evaluation of migration of normal MSCs through transwell porous membrane in the presence (black bar) or absence (white bar) of these EVs in the lower compartment of the transwell. (B) migration of MSCs after 72 hours stimulation with CSC-EVs (black bars) was evaluated by placing in the lower compartment of transwell, the tumor conditioned medium depleted of EVs or DMEM alone. Non-stimulated MSCs were used as control (white bars). (C) migration of MSCs after 2 week stimulus (black bars) versus tumor conditioned medium depleted of EVs or DMEM alone. Non-stimulated MSCs were used as control (white bars). Migration was assessed after 24 hours incubation. Statistical analysis was performed by ANOVA with Newman-Keuls multicomparison test: * indicates statistical difference to the control group maintained in the same experimental condition (P < 0.05; n = 5).

When unstimulated MSCs were plated in the upper chamber of transwell, no direct chemoattraction was observed by CSC-EVs (EVs) (Fig. 2A) or CSC conditioned medium (CTR TUM MED) (Fig. 2B, 2C) present in the lower chamber. When MSCs were pre-stimulated with CSC-EVs for a short time (72 h) and then challenged with CSC conditioned medium, a slight but not significant increase in migration was observed (STI TUM MED) (Fig. 2B). In contrast, when MSCs were pre-stimulated for 2 weeks with CSC-EVs, a significant increase in MSC chemo-attraction towards CSC conditioned medium was observed (STI TUM MED) (Fig. 2C).

### Changes in the expression of migration-related genes in MSC stimulated with CSC-EVs

**Figure 3:**
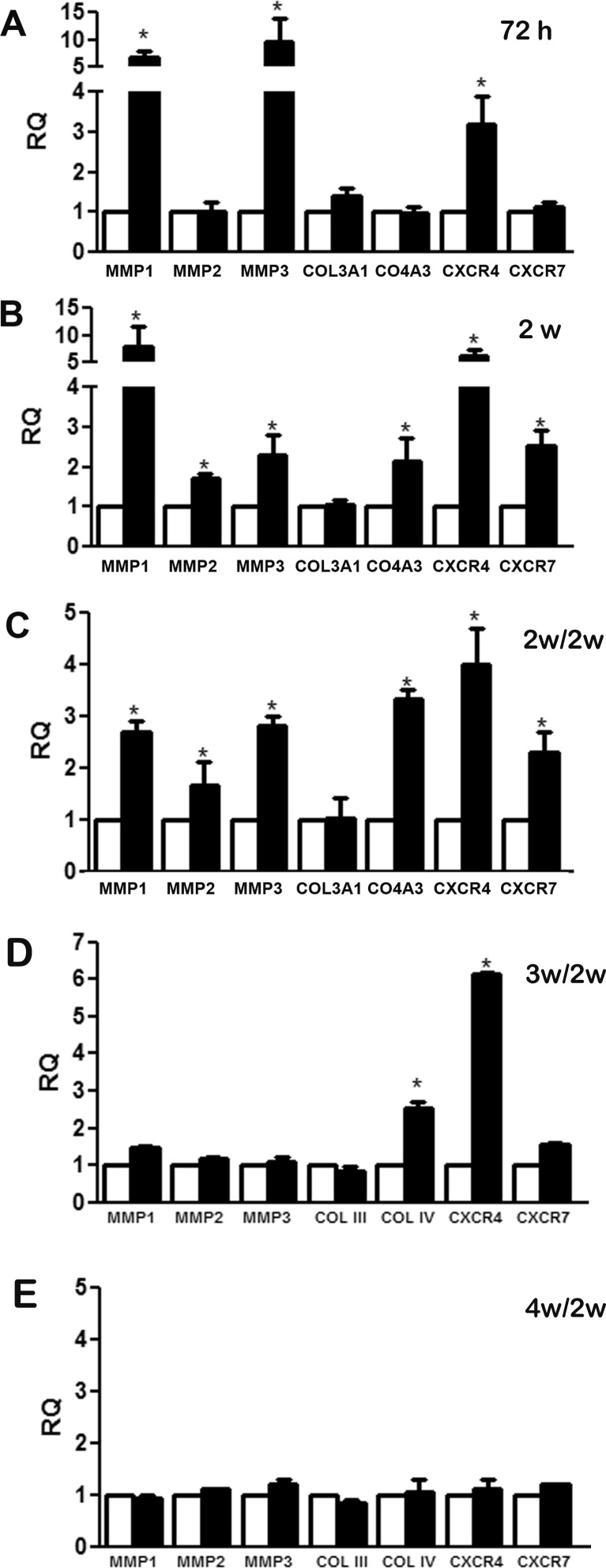
Changes in gene expression modulated by CSC-EV stimulation The expression of a group of selected genes associated with migration process was measured after CSC-EV stimulation. White bars represent non-stimulated MSCs as control group, black bars indicate CSC-EV stimulated MSCs. (A) modulation of gene expression in MSCs stimulated for 72 h. (B) modulation of gene expression in MSCs stimulated for 2 weeks. (C) modulation of gene expression in MSCs cultured for 2 additional weeks, (D) 3 additional weeks and (E) 4 additional weeks in the absence of CSC-EVs after 2 week stimulation. Data are expressed as RQ, normalized to GAPDH. Statistical analysis was performed by t-test: * indicates statistical difference in respect to control group (P < 0.05; n = 5).

The increase of MSC migration led us to investigate the modulation of genes related to the migration process such as matrix metalloproteinases (*MMP1*, *MMP2* and *MMP3*), collagens (*COL3A1* and *COL4A3*) and *CXCR4* and *CXCR7*. The analyses were performed in MSC stimulated with CSC-EVs for 72 hours or 2 weeks (Fig. 3A and 3B, respectively). After 72 hours of stimulus, the expression of *MMP1*, *MMP3* and *CXCR4* were significantly increased (black bars) when compared with unstimulated control MSCs (white bars). After 2 week-stimulation, a significant increase of *MMP1*, *MMP3*, *CXCR4, MMP2*, *COL4A3* and *CXCR7* was observed (Fig. 3B). To determine if the changes in gene expression were maintained in absence of persistent CSC-EV stimulation, we kept the 2 week-stimulated MSCs in culture for 2 additional weeks in normal medium without stimulus (Fig. 3C). The results obtained demonstrated the persistence of phenotypic changes observed in stimulated MSCs. Analysis of longer periods (3 and 4 weeks after MSC stimulation) showed a reversal expression of altered genes (Fig. 3D and 3E).

### CSC-EV-stimulated MSCs induced *in vitro* angiogenesis and tumor migration

**Figure 4:**
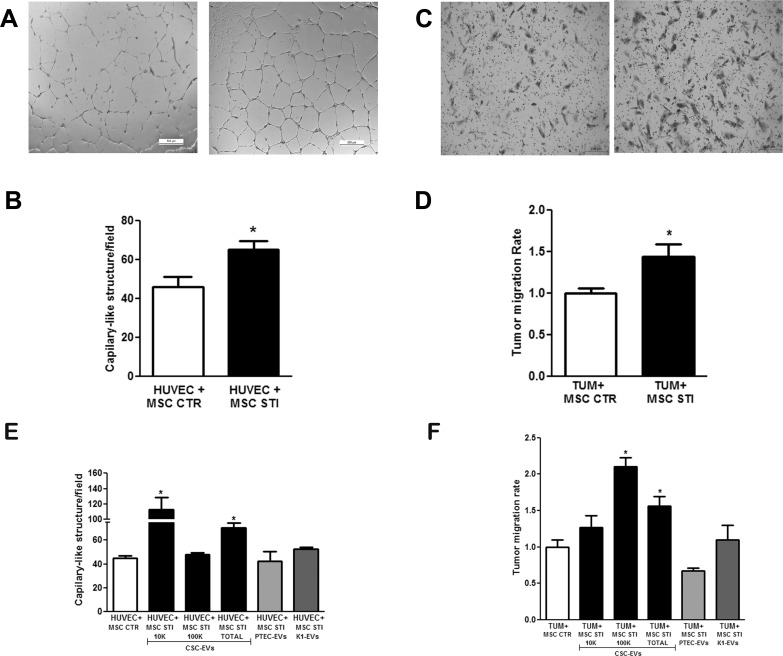
Effect of CSC-EV stimulation of MSCs on their ability to promote tumor migration and angiogenesis Experiments were performed using 2 week stimulated MSCs. (A) representative light microscopy images of vessel-like structures formed by HUVEC in co-culture for 24 hours with non-stimulated MSCs (left panel) or stimulated MSCs (right panel) (original magnification × 10). (B) quantification of vessel-like structures formed by HUVEC in co-culture with non-stimulated MSCs (white bars) and stimulated MSC (black bars). (C) representative light microscopy images of renal tumor cell migration after 24 hours co-cultured with non-stimulated MSCs (left panel) or stimulated MSCs (right panel) (original magnification × 10). (D) quantification of migration rate of K1 renal tumor cells after 24 hours of co-culture in transwell with non-stimulated MSCs (white bar) or stimulated MSCs (black bar). (E) comparison between CSC-EV fractions (10K, 100K and Total) and EVs derived from proximal tubular epithelial cells (PTEC) and K1 cells stimulated MSCs to induce formation of capillary-like structures. (F) comparison among the capacity of MSCs stimulated by CSC-EV, PTEC-EV or K1-EV fractions to stimulate tumor migration. Each condition is indicated in the abscissa. Statistical analysis was performed by t-test: * indicates statistical difference to the control group (P < 0.05; n = 4).

To evaluate whether MSCs stimulated for 2 weeks with CSC-EVs promoted angiogenesis, MSCs were plated in the upper transwell chamber and human umbilical vein endothelial cells (HUVEC) in the lower. As shown in Fig. 4A and 4B, CSC-EV-stimulated MSCs significantly increased the *in vitro* vessel-like formation by HUVEC (HUVEC + MSC STI).

To evaluate whether MSCs stimulated for 2 weeks with CSC-EVs promoted renal carcinoma (K1) cell migration, MSCs were plated in the lower transwell chamber and K1 cells in the upper. CSC-EV-stimulated MSCs significantly increased the migration of tumor cells in respect to control unstimulated MSCs (TUM + MSC STI) (Fig. 4C and 4D).

The specificity of MSC changes promoted by CSC-EVs were evaluated by comparing the effects of EVs derived from renal carcinoma K1 cells and non-tumorigenic cells renal proximal tubular epithelial cells (PTEC) on MSC using a 2 week-stimulus protocol. MSCs stimulated by PTEC-EVs did not induce angiogenesis (HUVEC+MSC STI PTEC-EVs) or tumor migration (TUM+MSC STI PTEC-EVs) (Fig. 4E and 4F). EVs derived from K1 cells induced a slight but not statistically significant increase in tumor migration (TUM+MSC STI K1-EVs) and angiogenesis (HUVEC+MSC STI K1-EVs) (Fig. 4E and 4F).

To evaluate which EV fraction induced MSC angiogenic and migratory properties, we isolated by differential ultracentrifugations, 10K and 100K EV fractions from CSC-EVs. When the two fractions were compared, we found that the MSCs stimulated with 10K fraction was more effective in inducing angiogenesis (HUVEC+MSC STI 10K CSC-EVs), whereas MSCs stimulated with 100K fraction mostly stimulated tumor migration (TUM+MSC STI 100K CSC-EVs) (Fig. 4E and 4F).

### Cytokines secreted by CSC-EV-stimulated MSCs

**Figure 5:**
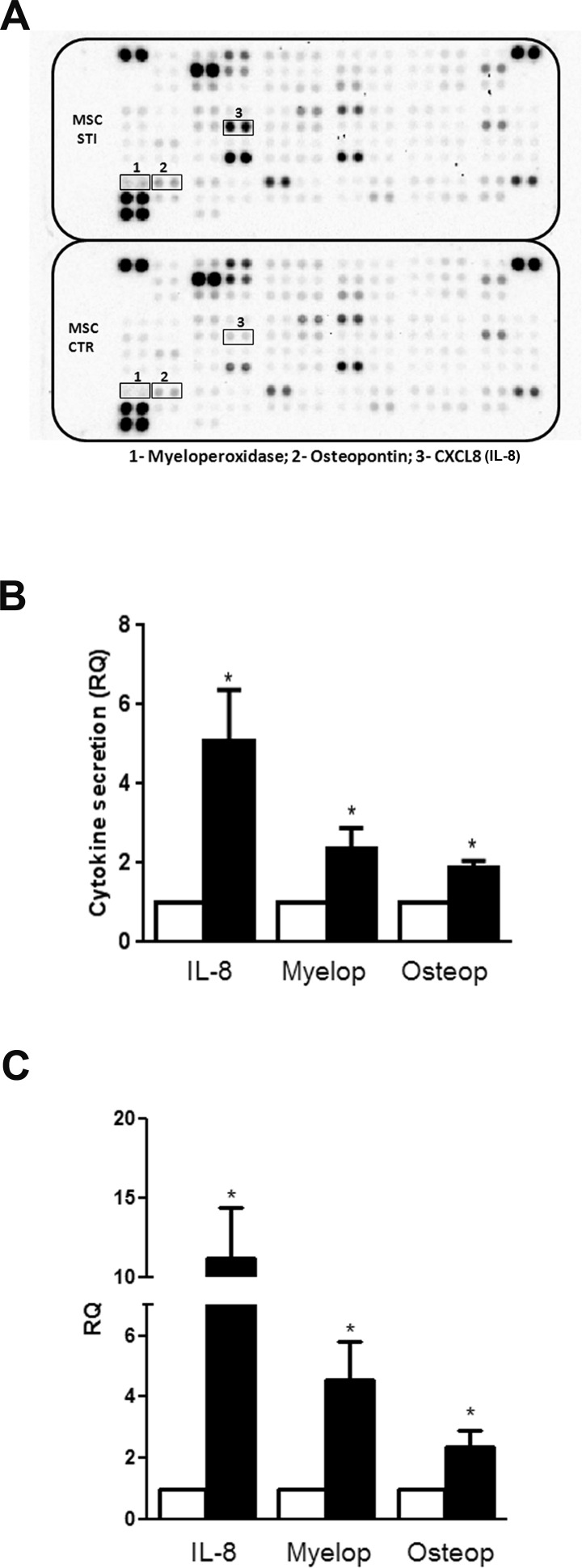
Alteration in cytokine secretion by MSCs after CSC-EV stimulation (A) representative cytokine array performed with supernatant of non-stimulated MSCs (lower panel) and stimulated MSCs (upper panel). The three molecules that presented a consistent change in all experiments performed are identified by black box (1-Myeloperoxidase; 2-Osteopontin; 3-IL-8). (B) relative quantification of the spots of the three secreted cytokines. White bars indicate non-stimulated MSC supernatants and black bars represent stimulated MSC supernatants. (C) gene expression of myeloperoxidase, osteopontin and IL-8 in 2 weeks stimulated MSCs (black bars). Non-stimulated MSCs were used as control (white bars). Data are expressed as RQ, normalized to GAPDH. Statistical analysis was performed by t-test: * indicates statistical difference to the control group (P < 0.05; n = 3).

Cytokine array analysis was performed in cell free supernatants of unstimulated and 2 week-stimulated MSCs to observe variation in the secreted molecules (Fig. 5A and 5B). From the group of cytokines evaluated, three molecules were consistently increased in the supernatant of stimulated MSCs (black bars) in all the experiments performed: IL-8, Myeloperoxidase (Myelop) and Osteopontin (Osteop). To determine if the increase in the secretion of these cytokines was due to a modulation at transcriptional level, we performed gene expression experiments. The results showed that the enhancement in the cytokine release by CSC-EV-stimulated MSCs (black bars) was due to an increase in their gene transcription (Fig. 5C).

### CSC-EV-stimulated MSCs supported *in vivo* tumor growth

**Figure 6:**
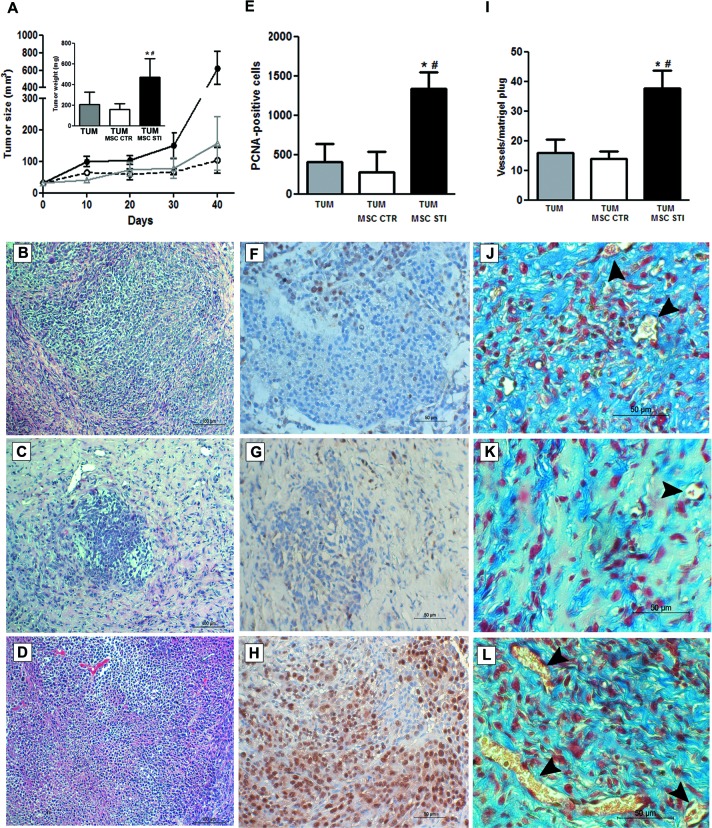
Effects of CSC-EV stimulated MSCs on *in vivo* tumor growth Experiments were performed in SCID mice that were divided in three groups (6 mice per group): subcutaneous injection of 1×10^6^ K1 tumor cells (TUM); subcutaneous injection of 1×10^6^ K1 tumor cells with 5×10^5^ non-stimulated MSCs (TUM MSC CTR); subcutaneous injection of 1×10^6^ K1 tumor cells with 5×10^5^ 2 weeks stimulated MSCs. Tumor growth was evaluated for 40 days and posterior analysis were performed in 5 μm paraffin tumor sections. (A) measurement of tumor growth during 40 days. TUM = gray line; TUM MSC CTR = dotted black line; TUM MSC STI = black line. The inset indicates the tumor weight measured after 40 days and each condition is identified in the abscissa. Representative hematoxylin and eosin staining of sections from the three experimental conditions: (B) TUM condition. (C) TUM MSC CTR condition. (D) TUM MSC CTR STI condition. (E) quantification of PCNA-positive cells in 10 random fields for each section at ×20 magnification. Each condition is indicated in the abscissa. Representative images of PCNA immunohistochemistry: (F) TUM condition. (G) TUM MSC CTR condition. (H) TUM MSC STI condition. (I) quantification of tumor vasculature measured by counting the number of erythrocyte-containing vessels in 10 random fields in trichrome stained tumor sections at original magnification × 20. Each condition is indicated in the abscissa. Representative images of tumor vasculature of the three groups: (J) TUM condition. (K) TUM MSC CTR condition. (L) TUM MSC STI condition. Statistical analysis was performed by ANOVA with Newman-Keuls multicomparison test: *, ^#^ indicates statistical difference to the TUM group and TUM MSC CTR group respectively (P < 0.05; n = 6).

The size of tumors formed by subcutaneous injection in SCID mice of K1 cells within Matrigel was significantly increased in the presence of 2 week CSC-EV-stimulated MSCs (TUM MSC STI) in respect to tumor alone (TUM) or tumor co-injected with unstimulated MSCs (TUM MSC CTR) (Fig. 6A). Such stimulation in tumor growth was confirmed by measurement of tumor weight (Fig. 6A inset). Histological analysis of tumors revealed a higher cell density of tumor epithelial cells in CSC-EV-stimulated MSCs in respect to unstimulated MSCs but not in respect to tumor alone (Fig 6B, 6C and 6D). Tumor with unstimulated MSCs showed a marked reduction of the epithelial component and an increase in the stroma. The size was slightly but not significantly reduced in respect to tumor alone.

PCNA staining by immunohistochemistry showed a higher proliferative rate in tumors co-injected with CSC-EV-stimulated MSCs (4.7 fold increase) than in tumor containing unstimulated MSCs (Fig. 6E, 6F, 6G, and 6H). In addition, a twofold increase in the number of vessels was observed in tumors co-injected with CSC-EV-stimulated MSCs in respect to tumor co-injected with unstimulated MSCs or tumor alone (Fig. 6I). Moreover, vessels present in tumors co-injected with CSC-EV-stimulated MSCs were larger in size in respect to tumor containing unstimulated MSCs (Fig. 6J, 6K, and 6L).

## DISCUSSION

Tumor development has been associated with the interactions between tumor cells and the surrounding stromal cells. This interaction has been, at least in part, ascribed to EV secretion [4-9]. MSCs are known to participate to stromal cell composition as they are recruited from bone marrow and engrafted within tumor [14]. The contribution of MSCs to tumor development and growth is still debated [15-22]. In the present study, we observed that renal CSC-derived EVs promoted pro-tumorigenic phenotype changes in MSCs that favor an increase of tumor vascularization and growth.

To reach and engraft the tumor, MSCs must be capable to sense and respond to tumor secretory stimuli [25, 26]. When MSCs were stimulated with CSC-derived EVs, they significantly increased the migratory capacity in response to tumor chemoattractive stimuli. Gene expression analysis revealed that CSC-EVs enhanced the expression of important genes related to migration process. *CXCR4* expression, which was increased after CSC-EV stimulation, is known to be involved in MSC migration through a SDF-1 concentration gradient secreted by tumor cells [27, 28]. Stimulated MSCs also presented an increase in *CXCR7* expression, a SDF-1 receptor associated with survival and paracrine actions of MSCs [29, 30]. In addition, several studies reported the high expression of these receptors in different tumor types and its association with angiogenesis, modulation of immune system and tumor invasion [31, 32].

The enhanced *MMP1*, *2* and *3* expression observed after stimulation with CSC-EVs suggested the capacity of MSCs to modulate matrix remodeling within tumor microenvironment. MMPs are proteolytic enzymes that are increased in the majority of human cancers and are associated with invasion, metastasis processes and with several other steps of tumor development like growth and angiogenesis [33]. The participation of stimulated MSCs in tumor matrix remodeling was also supported by the increase of *COL4A3* gene. This gene has been involved in regulation of cell adhesion, migration and metastasis in different tumor types [34-36]. The up-regulation of these genes (*MMP1*, *MMP2*, *MMP3*, *CXCR4/7*, and *COL4A3*) was maintained for at least 2 weeks after CSC-EV stimulation, suggesting persistent epigenetic alterations. Furthermore, one of the characteristics of MSCs stimulated with CSC-EVs, but not with EVs released from a total tumor cell population or from non-tumor cells, was the capacity to attract tumor cells and to promote angiogenesis, suggesting a role of MSCs in tumor spread. This observation suggests that EVs released from CSC rather than total tumor cell population play a central role in MSC pro-tumorigenic changes.

Communication between CSCs and MSCs was not unidirectional. In fact, MSCs, after CSC-EVs incorporation, changed their secretory profile increasing IL-8, osteopontin (OPN) and myeloperoxidase (MPO) production. MPO is known to be involved in oxidative stress response in tumors [37]. MPO has also been shown to be involved in the anti-apoptotic process by converting nitric oxide (NO) produced by inducible nitric oxide synthase (iNOS) into NO^+^ that promotes a S-nitrosylation of caspase-3, inhibiting its activity [38]. This alternative role of MPO may represent a mechanism by which MSCs support tumor development. IL-8 is a well described chemokine that plays an important role in tumor niche communication [39]. IL-8 can mimic the vascular endothelial growth factor (VEGF) and acts on endothelial cells enhancing their proliferation and survival [40]. In addition, IL-8 can be involved in the increase of tumor proliferation. In fact, this chemokine is associated with activation of signaling pathways like mitogen-activated protein kinase (MAPK), focal adhesion kinase (FAK) and Src-kinase pathway [41, 42]. OPN is another important molecule in the crosstalk between MSCs and cancer cells. OPN is highly expressed in tumor stroma and it is involved in signaling regulation processes linked to angiogenesis, metastasis and tumor growth in different tumors [43]. The role of OPN in clear cell renal cell carcinoma has been shown to be mediated by activation of nuclear factor-kappa B (NF-κB) promoting tumor progression by protecting cells from apoptosis [44]. OPN also presents an autocrine action on MSCs by stimulating FAK and ERK signaling pathways via β1-integrin activation. This resulted in the reduction of MSC stiffness, in the increase of motility and consequently in MSC migration [45].

In our experimental condition, we observed that MSCs stimulated by CSC-EVs were capable to support *in vivo* tumor growth by increasing proliferation and vascularization. In contrast, unstimulated MSCs were unable to promote tumor growth suggesting the need of MSC pre-conditioning by tumor microenvironment. In fact, several studies reported the role tumor-EVs in the communication with surrounding cells creating an environment favorable to tumor growth [6-10]. Experiments performed by Wysoczynski and Ratajczak showed that EVs secreted by lung cancer support angiogenesis by promoting changes in stromal cells, increasing the expression of several pro-angiogenic factors (as IL-8, VEGF, OSM, MMP9) [46]. CSCs may represent an important part in the establishment of this tumor environment through the secretion of EVs. The effects of CSC-EVs on MSCs point on the role of these vesicles in tumor communication with stromal surrounding cells. Such role of EVs in tumor crosstalk, especially with MSCs, brings important information for anti-tumor therapies. The anti-tumor potential of MSCs may depend on type or even stage of tumor development. Whereas, naive MSCs may display an anti-tumor activity [15-17], MSCs pre-conditioned by tumor EVs may change their phenotype and promote tumor growth. A possible strategy to overcome such condition is to pharmacological inhibit release of tumor EVs before the administration of MSCs, thus preventing their detrimental effects [46, 47].

In conclusion, the results of the present study suggest the importance of EVs in tumor-MSC interaction. In particular, CSCs can alter MSC phenotype through secretion of EVs. Altered MSCs became more responsive to tumor chemoattractive stimuli and supportive to tumor cell migration and proliferation, and may favor tumor vascularization. The phenotypic changes were maintained even after removal of stimulation, suggesting a persistent change in MSC phenotype.

## METHODS

### Cell culture

K1 cell lines were previously isolated from renal carcinoma cells and characterized. These cells are tumorigenic cells positive for CD40 and CD154 markers [48]. K1 cells were cultured in Roswell Park Memorial Institute (RPMI)-1640 (Life Technologies) supplemented with 10% heat-inactivated fetal calf serum (FCS) (Sigma). Three CSC (CD105^+^) clones previously isolated and characterized were used for the experiments. The CD105^+^ cells were isolated from human renal carcinomas and presented clonogenic ability, stem cells markers, ability to differentiate and can be cultured in suspension. In addition, CSC clones (1×10^5^ cells) were able to generate heterogenic tumors [24]. CSCs were cultured in Dulbecco's Modified Eagle's Medium (DMEM) supplemented with hydrocortisone 2.5 nM, insulin-transferrin-selenium 1×, epidermal growth factor 1.7 nM and 5% FCS. As previously reported, CSCs were CD105 positive and negative for the endothelial or haematopoietic markers CD31, VEGF receptor 2 and CD45. Moreover, they showed cancer stem cell properties as expression of stem cell markers and absence of markers of differentiation. In addition, CSCs displayed the ability to grow in spheres and to initiate tumors and generate serially transplantable tumors with a number of cells as low as 100 cells/mice [6].

Angiogenesis *in vitro* was performed with HUVECs that were isolated and characterized as previously described [49].

Human MSCs were obtained from Lonza (Basel, Switzerland), cultured in the Mesenchymal Stem Cells Basal Medium (MSCBM, Lonza) and characterized as previously described [50]. All the experiments were performed with cells maintained in culture until six passages. The MSCs were characterized by FACS analysis and were positive for CD105, CD73, CD44, CD90, CD166, CD146 and HLA class I; and negative for hematopoietic markers (CD45, CD14 and CD34) and for costimulatory molecules (CD80, CD86 and CD40). Moreover, osteocytic, adipogenic and chondrogenic differentiation capabilities were also assessed [48]. Immortalized renal tubular epithelial cells (PTEC) were purchased from ATCC (HK-2 cells) and cultured with DMEM supplemented with 10% FCS.

### Isolation of EVs and incorporation by MSCs

The CSCs, K1 and PTEC were cultured overnight in RPMI medium without phenol red (Life Technologies). The supernatants were centrifuged at 300 g for 20 min to remove debris and subsequently centrifuged at 100,000 g (Beckman Coulter Optima L-90K ultracentrifuge) for 1 hour at 4^o^ C. In selected experiments CSC-EVs were separated in two fractions by a centrifugation at 10,000 g (10K) for 1 hour at 4^o^ C and followed by ultracentrifugation at 100,000 g (100K) for 1 hour at 4^o^ C of supernatants deprived of the 10K fraction. Vesicles were studied as previously reported [6] by cytofluorimetric analysis. CSC-EVs were CD105+ and expressed CD44 and adhesion molecules such as α5 and α6 integrins. The amount and size of CSC-EVs were determined by NanoSight. CSC-EVs ranged from 30 nm to 390 nm, with a mean value of 181 nm (Supplementary Figure 1). The amount of particles with size below 30 nm was 0.9 ± 0.5 % and was subtracted from total counts. Limulus test for LPS contamination was negative (Charles River Laboratories, Inc., Wilmington, MA, USA). In selected experiments, CSC-EVs were stained in red with Vybrant^®^ Dil (Molecular Probes) [16] to evaluate their incorporation by MSCs. The stained CSC-EVs were cultured with MSCs with a ratio of 5×10^4^ vesicles/stimulated cell for different periods of time (6, 24, 48, 72 h). MSCs were stained in green with Syto^®^ RNA select (Molecular Probes). The incorporation of CSC-EVs by MSCs was analyzed by confocal microscopy.

Functional experiments were performed under two protocols of stimulation: MSCs were incubated with CSC-EVs (5×10^4^ vesicles/stimulated cell) for 72 hours (short stimulation); or MSCs were repeatedly stimulated every 5 days with CSC-EVs (5×10^4^ vesicles/stimulated cell) for 2 weeks (long stimulation), performing a total of 3 stimuli.

### MSC and tumor cell migration

The migration assay was performed using a transwell system (Becton Dickinson) with 8 μm pore size. To observe the changes in the migratory capacity, unstimulated or CSC-EV-stimulated MSCs were plated in the upper compartment of a transwell. Based on experimental conditions, the inferior compartment was fulfilled with CSC-EVs (to determine vesicles chemoattraction) or EV-deprived CSC conditioned medium (to analyze the MSC migration towards CSC secreted factors) or medium alone. After 24 hours incubation, non-migrated cells were removed from the membrane upper surface with cotton swabs. Migrated cells were then fixed with absolute methanol and stained with 0.2% crystal violet solution. MSC migration rate was determined by counting the number of cells contained in the photos of 5 different fields at original magnification × 10.

To analyze the capacity of stimulated MSCs to induce tumor migration, K1 cells (2×10^4^ cells) were co-cultured for 24 hours with unstimulated or with CSC-EV-stimulated MSCs (ratio 1:1). The analyses of tumor migration were performed as previously described [51].

### *In vitro* angiogenesis assay

HUVECs (2×10^4^ cells) were plated into 24-well plates coated with growth-factor depleted Matrigel^®^ (Corning) and co-cultured with unstimulated or 2 week stimulated MSCs (2×10^4^ cells) in a transwell system of 0.4 μm pore size (Becton Dickinson) for 24 h. The MSC angiogenic potential was determined by the number of vascular-like structures formed by endothelial cells.

### RNA extraction and real-time quantitative polymerase chain reaction (qRT-PCR)

RNA was isolated with Trizol^®^ Reagent (Life Technologies) from unstimulated, 72 h-stimulated, 2 week-stimulated MSCs or 2 week-stimulated MSCs maintained after washing for 2 additional weeks in culture without CSC-EVs. The obtained RNA was quantified by spectrophotometry (Nanodrop ND-1000) and 200 ng total RNA of each sample condition was reversely transcribed to cDNA using High Capacity cDNA Reversion Transcription kit (Life Technologies). The mRNA expression in MSCs was assessed by qRT-PCR using Power SYBR^®^ Green PCR Master Mix (Life Technologies), performed in a 96-well StepOne Real-Time System (Applied Biosystems). The sequence-specific oligonucleotide primers were all obtained from MWGBiotech AG, Ebersberg, Germany (www.mwg-biotech.com). The relative gene expression was measured using *GAPDH* as housekeeping gene. List of primers used is reported in Supplementary Table 1.

### Secreted cytokine assay

To identify changes in the pattern of molecules secreted by MSCs, the supernatants of unstimulated and 2 week-stimulated MSCs cultured in RPMI overnight were collected and centrifuged to remove cellular debris. Experiments were then performed using a Human XL Cytokine Array Kit (Proteome Profiler™ Array - R&D Systems) according to the manufactory protocol. Quantification was performed by ChemiDoc™ XRS+ System and data analyses were performed using Quantity One® Analysis Software (Bio-Rad).

### *In vivo* tumor growth analysis

All animal experiments have the approval of the Ethics Committee of Turin University. To evaluate the *in vivo* effects on tumor growth of MSCs stimulated with CSC-EVs for 2 weeks, SCID mice were divided in 3 groups (n=6 for each group): Group 1 was subcutaneously injected with renal carcinoma (K1) cells (1×10^6^ cells); Group 2 was subcutaneously injected with K1 cells (1×10^6^ cells) mixed with unstimulated MSCs (0.5×10^6^ cells); Group 3 was subcutaneously injected with K1 cells (1×10^6^ cells) mixed with MSCs (0.5×10^6^ cells) stimulated for 2 weeks with CSC-EVs. Prior to the injection harvested cells were counted and mixed in 250 μL of cold DMEM. The cells were then added to 250 μL of growth-factor depleted Matrigel® at 4°C and subcutaneously injected using a 1-ml syringe with 26-gauge needle. Tumor volume was measured with a caliper and calculated using the formula: v= L × l^2^ × 0.5 where “L” indicates the large diameter and “l” indicates the small diameter as previously described [18]. After 40 days, mice were sacrificed and tumors were weighted and then fixed and embedded in paraffin. Histological analysis was performed by hematoxylin/eosin staining. Immunohistochemistry analyses were performed in 5 μm paraffin tumor sections. Cell proliferation was measured by overnight incubation with mouse anti-PCNA antibody (sc-56, Santa Cruz) at 4°C, followed by 1 hour incubation at room temperature with Immunopure^®^ goat anti-mouse peroxidase conjugate secondary antibody (31430, Thermoscientific). The substrate 3,3′ –diaminobenzidine (DakoCytomatation) was used for color development. The sections were then counterstained with hematoxylin. PCNA positive cells were counted in 10 random fields for each section at ×200 magnification. Tumor vascularization was measured by counting the number of erythrocyte-containing vessels in 10 random fields of trichrome stained tumor sections at original magnification × 20 per each sample.

### Statiscal analyses

Statistical analyses were performed using t-test or ANOVA followed by the Newman-Keuls multicomparison test when appropriate. Statistical significance was set at P < 0.05. Data were analyzed using the GraphPad Prism 5.0 Demo program.

## SUPPLEMENTARY MATERIAL, FIGURES, TABLES


