# Supplementary material for: Extracellular vesicles derived from renal cancer stem cells induce a pro-tumorigenic phenotype in mesenchymal stromal cells
Source: Oncotarget. 2015 Mar 10;6(10):7959–69. doi: 10.18632/oncotarget.3503 (PMC4480728; doi:10.18632/oncotarget.3503)
Supplement: Supplementary file 1 [file oncotarget-06-7959-s001.pdf]

Extracellular vesicles derived from renal cancer stem cells induce a pro-tumorigenic phenotype in mesenchymal stromal cells

Supplementary Material

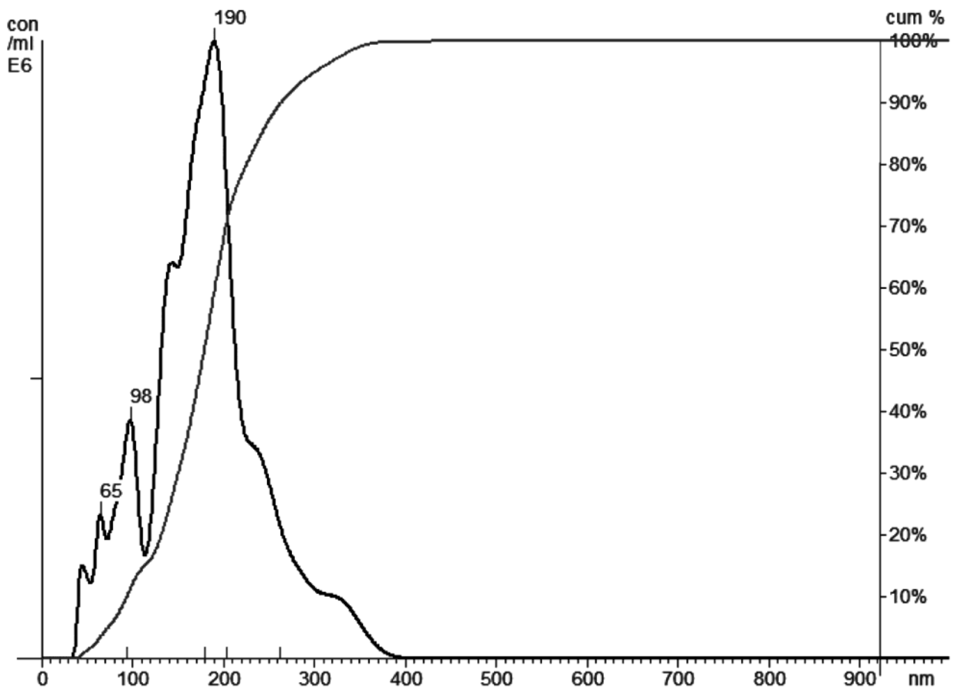

Supplementary Figure 1

Representative image of Nanosizer analysis of CSC-EV: the peaks show the relationship between particle distribution (left Y axis) and particle size (X axis); the curve describes the correlation between cumulative percentage distribution of particles (percentile in right Y axis) and particle size (X axis). Mean size and particle concentration values were calculated by the Nanoparticle Tracking Analysis (NTA) software that allows analysis of video images of the particle movement under Brownian motion captured by Nanosight LM10 and calculation of the diffusion coefficient, sphere equivalent and hydrodynamic radius of particles by using the Strokes-Einstein equation.

Supplementary Table 1: List of primers for the qRT-PCR experiments.

Human genes	Forward	Reverse
**Human matrix metallopeptidase 1 (*MMP1*)**	5'- CCAACAATTTCAGAGAGTACAACTTACAT -3'	5'- TGAAGGTGTAGCTAGGGTACATCAAA -3'
**Human matrix metallopeptidase 2 (*MMP2*)**	5'-ACCAAGGGTACAGCCTGTTCCT -3'	5'- CTGGGACAGACGGAAGTTCTTG -3'
**Human matrix metallopeptidase 3 (*MMP3*)**	5'- GCTGTTTTTGAAGAATTTGGGTTCT -3'	5'- TTCAACAATTAAGCCAGCTGTTACTC -3'
**Collagen, type IV, alpha 3 (*COL4A3*)**	5'-TCATCATGTTCACAAGTGCAGGT-3'	5'- AGTAGTTGCACGTTCCTCTTCCAT -3'
**Collagen, type III, alpha 1 (*COL3A1*)**	5'- CAGGAAGCTGTTGAAGGAGGAT -3'	5'- CGGATCCTGAGTCACAGACACA -3'
**Chemokine (C-X-C motif) receptor 4 (*CXCR4*)**	5'- CAGTGGCCGACCTCCTCTT -3'	5'- CAGTTTGCCACGGCATCA -3'
**Atypical chemokine receptor 3 (*CXCR7*)**	5'- GAGACCACTTCATGCCTTGCA -3'	5'- CTAGACTTTCCTCTTTGCCTTTCG -3'
**Interleukin 8 (*IL8*)**	5'- CTGTGTGAAGGTGCAGTTTTGC -3'	5'- CAGTGTGGTCCACTCTCAATCAC -3'
**Myeloperoxidase (*MPO*)**	5'- TGGTCCAGATCATCACTTACCG -3'	5'- GTCATTGTAGGAACGGTACGTGG -3'
**Secreted phosphoprotein 1 *(OPN)***	5'- CCTGCCAGCAACCGAAGTT -3'	5'- TGTAGCATCAGGGTACTGGATGTC -3'
**Glyceraldehyde 3-phosphate dehydrogenase (*GAPDH)***	5'-TGGAAGGACTCATGACCACAGT-3′	5′-CATCACGCCACAGTTTCCC-3′
